# Recent Advances in the Aptamer-Based Electrochemical Biosensors for Detecting Aflatoxin B1 and Its Pertinent Metabolite Aflatoxin M1

**DOI:** 10.3390/s20113256

**Published:** 2020-06-08

**Authors:** Hadi Beitollahi, Somayeh Tajik, Zahra Dourandish, Kaiqiang Zhang, Quyet Van Le, Ho Won Jang, Soo Young Kim, Mohammadreza Shokouhimehr

**Affiliations:** 1 Environment Department, Institute of Science and High Technology and Environmental Sciences, Graduate University of Advanced Technology, Kerman 76315117, Iran; h.beitollahi@yahoo.com (H.B.); z.dourandish2017@gmail.com (Z.D.); 2 Research Center for Tropical and Infectious Diseases, Kerman University of Medical Sciences, Kerman 7616913555, Iran; 3 Jiangsu Key Laboratory of Advanced Organic Materials, Key Laboratory of Mesoscopic Chemistry of MOE, School of Chemistry and Chemical Engineering, Nanjing University, Nanjing 210023, Jiangsu, China; kaiqiangzhang126@126.com; 4 Institute of Research and Development, Duy Tan University, Da Nang 550000, Vietnam; 5 Department of Materials Science and Engineering, Research Institute of Advanced Materials, Seoul National University, Seoul 08826, Korea; hwjang@snu.ac.kr; 6 Department of Materials Science and Engineering, Korea University, 145, Anam-ro Seongbuk-gu, Seoul 02841, Korea

**Keywords:** electrochemical biosensors, aflatoxin B1, aflatoxin M1, aptamer

## Abstract

The notable toxicological impacts of aflatoxin B1 (AFB1) and its main metabolite, aflatoxin M1 (AFM1), on human being health make the evaluation of food quality highly significant. Due to the toxicity of those metabolites—even very low content in foodstuffs—it is crucial to design a sensitive and reliable procedure for their detection. Electrochemical aptamer-based biosensors are considered the most encouraging option, based on multi-placed analysis, rapid response, high sensitivity and specificity. The present review specifically emphasizes the potential utilization of the electrochemical aptasensors for determining the AFM1 and AFB1 with different electrodes.

## 1. Introduction

It is widely accepted that mycotoxins are fungal toxic metabolites able to contaminate food materials. In fact, experts are currently attracted by these metabolites because of the presence of mycotoxin in the food products which results in pertinent negative impact on the humans’ health and environment. Notably, the immunosuppressive, teratogenic, nephrotoxic, mutagenic and carcinogenic are their major impacts. Aflatoxins (AFs) as one of the highly poisonous compounds obtain intensive attention which are able to contaminate multiple foodstuffs like corn, cotton seeds, peanuts, almond, nuts, pistachio, figs, spices, cheese as well as milk in diverse beverages and foodstuffs. Moreover, they have essential resistance even at increased temperature, and hence can tolerate the cooking procedures [1,2,3].

According to the studies, AFs are considered to be the key toxic secondary metabolites of a number of *Aspergillus* molds, such as *Aspergillus flavus*, rare *Aspergillus nomius* and parasitic *Aspergillus*. Among multiple kinds of aflatoxins (B1, B2, G1, G2 and M2), AFB1 is found as one of the prime carcinogenic compounds by the International Agency for Research on Cancer (IARC) [4].

The U.S. Department of Agriculture and the U.S. Food and Drug Administration have established an “actionable” level of 15–20 ppb of AFs in animal feed products. In 1973, the European Economic Community established legislation on maximum permitted levels of AFBl in different types of feedstuffs. The legislation has been frequently amended since then. The European Community levels are more restrictive; four micrograms kg^−1^ total aflatoxin in food for human consumption is the maximum acceptable limit, which is the strictest in standard worldwide. Human foods are allowed 4–30 ppb aflatoxin, depending on the country involved [5,6].

Moreover, it is a mycotoxin with powerful teratogenic and mutagenic features. In addition, analyses revealed the ability of AFB1 in inducing primary liver, stomach and lung cancers. Furthermore, AFB1 is one of the experimental hepatocarcinogens which has high risk in the multifactorial etiology of the humans’ hepatic cellular cancer. Hence, median lethal dose (LD50) of AFB1 is equal to 0.36 mg kg^−1^ (body weight) [7,8].

Therefore, in the case of ingestion of AFB1, as the most poisonous aflatoxin, by the cows via a polluted foodstuff, the metabolite would be transformed into AFM1 via an enzymatic hydroxylation of AFB1 at the 9a position and had a nearly overall conversion rate of 0.3–6.2%. In fact, AFM1 would be secreted in milk through the mammary glands of the dairy cows. A protein fraction of milk, particularly casein, binds AFM1 and in the case of the existence of the AFM1 in the raw milk; cheese prepared from this milk will contain AFM1. Studies indicated high toxicity and carcinogenicity of AFM1. Hence, IARC divided it as a group 1 human carcinogen. Moreover, the European Commission treats 0.5 to 50 ng mL^−1^ as the maximum residue level (MRL) for AFB1 and AFM1 in the edible foodstuffs and milk. However, due to the high poisonousness, determining and quantifying the sub-nanogram in each gram concentration of such toxins in the foodstuffs would be highly advised [9].

Based on the studies in the field, the widely applied techniques for determining AFM1 and AFB1 include the thin layer chromatography (TLC) [10], liquid chromatography coupled with the mass spectroscopy (LC-MS) [11], and the high-performance liquid chromatography (HPLC) [12]. Nonetheless, innate features related to the chromatographic procedures like the long and complex sample pretreatment techniques, costly instrumentations and necessity of the skillful technicians limited their extensive applications in the high-throughput and on-site analyses of the samples [13].

Therefore, experts in the field confirmed usefulness of the electrochemical biosensors to determine the food contaminants. In fact, they intensively investigated the electrochemical biosensor, particularly aptasensor, (on the basis of the strongly specific molecular recognition of antigens by aptamer) in terms of detecting diverse biomolecules, because of their inexpensiveness, simplification, higher sensitivity, portability, compatibility with the mass manufacturing and possible micro fabrication. Furthermore, in the case of the use of nanoparticles (NPs in the transducing segment of the aptasensor), the signals depict an effective enhancement [13,14]. Therefore, we reviewed the aptamer-based electrochemical biosensors that are designed to determine AFM1 and AFB1.

## 2. Aptamers

Aptamers are considered the single-stranded oligonucleotides (usually RNA or DNA) or peptides with the ability of binding to the respective targets with higher specificity and affinity as the same as the antigen–antibody interaction. Therefore, the selection procedure is well-known as the systematic evolution for the ligands via exponential enrichment (SELEX), which is discovered in 1990. In fact, the SELEX begins with a chemically synthesized random oligonucleotides library (up to 1015 distinct sequences). Additionally, the mentioned selection procedure can be categorized into three phases of binding, separation/partitioning and amplification, which will be iterated for obtaining nucleotides with greater binding abilities toward the intended targets. Following some cycles (usually 5 to 15), the sequences would be cloned and sequenced their binding affinity, secondary structure as well as the Gibbs free energy would be assessed for selecting aptamers with the increased specificity and affinity to the target molecule [15].

Antibodies or immunoglobulins (*I*_g_) are highly soluble serum glycoproteins involved in the defense mechanisms of the immune system. They can be divided into five classes based on their heavy chain constant region sequences, i.e., *I*_g_M, *I*_g_D, *I*_g_G, *I*_g_E and *I*_g_A. Antibodies are proteins with Y shape, with the ability of recognizing specific molecules, such as antigens, via the antigen-binding fragment variable region. Generally, an antigen has several epitopes which cause several antibodies formation, these are polyclonal antibodies. Antibodies have been known for more than three decades and are proved to be invaluable tool for rapid and advanced diagnostics. Although, antibodies are used routinely in most of diagnostic tests as biorecognition elements, however, their high cost of production, shorter shelf life, requirement of animals for production, stability issues and batch-to-batch variations have significant drawbacks on the field of diagnostics [16,17].

Unlike antibodies, aptamers can easily be regenerated for repeated use as aptamers can be denatured and refolded into a functional configuration for ‘n’ number of times. The derivatized nucleic acid aptamers are emerging as promising alternatives to monoclonal antibodies. Aptamers can also be used in a multiplex platform to detect multiple targets simultaneously, where antibody often fails due to its cross reactivity [18,19].

However, in comparison to the antibodies, the aptamers enjoy the features of the simplified construction and iterated utilizations. Moreover, they cannot tolerate the physical and chemical instability or potent immunogenicity. Therefore, experts in the field growingly utilize them as the recognition components in biosensors. Because of easy functionalization of the oligonucleotides, aptamers could be conjugated virtually to all types of nanomaterials. In addition, they enjoy higher selectivity and affinity, greater resistance to the harsh treatments without damage to bioactivity, reversible thermal denaturation, immobilization, regeneration and signaling. Consequently, they would be a promising option for designing aptasensors [20,21].

## 3. Electrochemical Techniques for AFs Detection

Electrochemical techniques were also applied to the determination of AFs content and AFs capacity. Electrochemical impedance spectroscopy (EIS) and voltammetry techniques are the most broadly used. EIS technique is low-cost, sensitive and relatively easy to use. EIS is a non-destructive steady state technique and can be used as an ideal tool to detect the dynamics of biomolecular interactions. EIS measurements are widely used in the field of electrochemical sensors and biosensors which are performed in the presence of a redox agent, to measure the molecular interactions of electrochemically inactive compounds taking place on the electrode surface for characterization and diagnostics as well as a quantitative detection method. In particular, EIS-based biosensors are well-suited to the detection of binding events occurring on the transducer surface since minute changes in analytes to a biosensor surface can be easily and rapidly detected. However, the high sensitivity of the method, being highly advantageous, can be also associated with nonspecific impedance changes that could be easily mistaken for specific interactions, that is, inability to discriminate between specific and nonspecific binding. Therefore, it is very necessary to improve its selectivity by modifying electrode surface with substance that can specially interact with analytes. Thus, seeking for a recognition element, such as aptamer to bind AFs specifically is the key factor for highly selective detection [22,23].

Voltammetry is a potentiodynamic technique, based on measuring the current arising from oxidation or reduction reactions at the working electrode surface, when a controlled potential variation is imposed.

Voltammetric techniques include various methods such as cyclic voltammetry (CV), differential pulse voltammetry (DPV) and square wave voltammetry (SWV). Cyclic voltammetry is often the first experiment performed in an electrochemical study of a compound, a biologic material or an electrode surface. It is effectively used in the fields of environmental electrochemistry, organic chemistry, inorganic chemistry and biochemistry. The effectiveness of CV results from its capability of rapidly observing the redox behavior over a wide potential range [24,25].

A pulse technique was proposed by Barker and Gardner to increase the sensitivity of the technique and to lower the detection limits for electroactive species. Differential pulse voltammetry is extremely useful for the determination of trace amounts of electroactive compound in food samples, pharmaceuticals and biologic fluids [26]. Square-wave voltammetry is a large amplitude differential technique in which a waveform is composed of symmetrical square waves. Excellent sensitivity in SWV is gained from the fact that net current is large compared to either forward or backward current, coupled with effective discrimination against the charging current. The peak currents obtained are about four times higher than the differential pulse response [27]. These are the most widely used techniques to study the kinetics of electrochemical reactions, determination of formal redox potentials, reversible and irreversible reactions of analytes under study.

## 4. Electrochemical Aptasensors for AFs Detection

It is widely accepted that sensor system fundamentally contains two sections: A signal transducer and recognition element. In the case of electrochemical sensors, transducer consists of electronic detection system as well as properly modified electrode substrate. In the case of electrochemical aptasensors, the modified layer frequently contains the organic molecules and or nanomaterials and is used as a support material linking the aptamer probes to substrate.

Notably, the electrodes applied in constructing the electrochemical aptasensors are commonly the diverse electrodes like the glassy carbon electrode (GCE), gold electrode, screen-printed electrode (SPE), etc. It is notable that the electronic detection system frequently involves a signal amplifier, display screen and processor. However, in a majority of the electrochemical aptasensors, immobilization of the aptamers is performed on an electrode substrate so that they are free in interacting with the intended analyte in the sample. Finally, the transducer is used to measure the interaction, which presents a signal proportional to the target analyte concentration in the sample [21]. Now, diverse electrode-based aptasensors utilized to determine AFM1 and AFB1 are presented.

### 4.1. GCE-Based Aptasensors

Experts in the field have extensively investigated the GCE due to respective wider potential ranges, chemical inertness, inexpensiveness and lower porosity. Therefore, they utilized the chemical, electrochemical, thermal and plasma pretreatments as well as mechanical polishing on the carbon electrodes before doing the electrochemical experiments. Moreover, treatments have been applied for producing a surface as free as possible from the contamination and activating the electrode towards the electron transfer [28].

The study conducted by Evtugyn nt al. designed electrochemical aptasensors based on GCE modified with the electropolymerized Neutral red (NR) and polycarboxylate macrocyclic ligands, through which the DNA aptamers were covalently binding to detect AFB1. It has been found that there is a covalent relationship between aptamer against AFB1 and NR label and the carrier carboxylic groups by carbodiimide binding. In fact, interacting with an analyte caused lower cathodic peak current of the probe gauged by CV and greater resistance of the electron transfer as recognized by EIS. Moreover, limit of detection (LOD) is equal to 0.05 nM for EIS and 0.1 nM for CV. Thus, aptasensor provided the ground for detecting AFB1 in the peanuts, soy sauce, cashew nuts and white wine with the recoveries between 85% and 100% [1].

Additionally, Geleta et al. presented the reduced graphene oxide/molybdenum disulfide/polyaniline@ gold NPs (AuNPs)-based electrochemical aptasensor (called RGO/MoS_2_/PANI@AuNPs/Apt) to detect AFB1. In the next stage, they modified GCE via RGO/MoS_2_/PANI nanocomposites coated by chitosan (Cs) film, and then AuNPs bound to immobilize the AFB1 aptamers. However, in the presence of AFB1, the AFB1 binding resulted in the conformation changes in the immobilized aptamer on the surface of electrode, reducing the electron transfer within [Fe(CN)_6_] ^3−/4−^ redox couple present in solution to GCE surface. Hence, the peak current change of RGO/MoS_2_/PANI@AuNPs/Apt could be readily controlled via DPV. Based on the optimum condition, the as-developed RGO/MoS_2_/PANI@AuNPs/Apt exhibited wider linear ranges between 0.01 and 1.0 fg/mL and a notably LOD (3σ) = 0.002 fg/mL. Finally, AFB1 in the spiked wine samples has been analyzed, which confirms RGO/MoS_2_/PANI@AuNPs/Apt functionality [29].

The study conducted by Smolko et al. designed an aptasensor for the strongly sensitive detection of AFM1 based on the GCE coated with the polymeric NR dye [5]. In the presence of AFM1, the cathodic peak current associated with NR conversion decreases. Moreover, AFM1 caused the charge transfer resistance increasing, which has been measured by EIS. Based on the optimum conditions, it could be determined in the range between 5 and 120 ng/L AFM1 into the standard solution with LOD equal to 0.5 ng/L. furthermore, validity of the aptasensor has been confirmed in the spiked samples of the sheep and cow milk. Finally, reliable detection of 40–160 ng/kg mycotoxins is observed [30].

**Figure 1:**
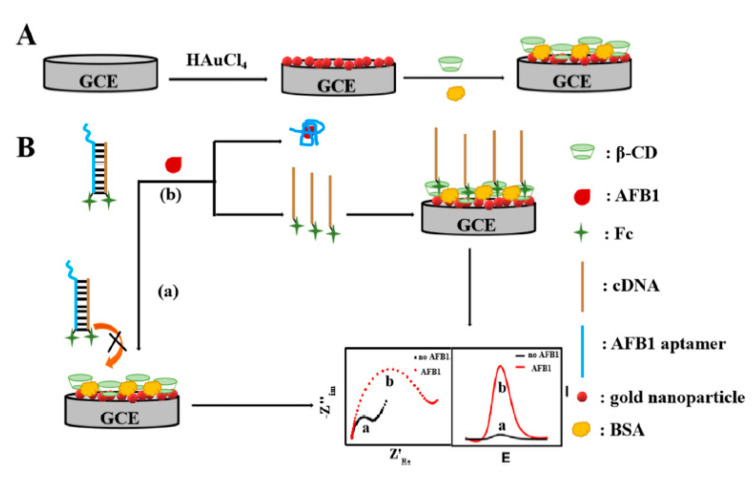
A schema of the construction process of electrochemical aptasensor for AFB1 (**A**) and its detection approach (**B**). Reprinted with permission from [14]. Copyright 2019, Elsevier.

In their study, Wu et al. presented an electrochemical aptasensor to detect AFB1 on the basis of the smart host guest recognition between the ferrocene (Fc) and β-cyclodextrin (β-CD). The host–guest complexes are formed between cyclodextrin and other species, the cyclodextrin is a host. To improve the electron transfer feature of the bare GC electrode, Au NPs have been deposited on the surface of GC. Moreover, electrochemical polymerizing β-CD has been performed on the AuNPs/GC electrode surface by CV. In fact, without AFB1, hybridization of Fc-labeled probe DNA (Fc-cDNA) with the Fc-labeled aptamer is observed. Therefore, 2 ferrocene molecules approximated to each other and could not be identified by β-CD. Upon the capture of the aptamer from the hybridization structure by AFB1, Fc-cDNA is released and attached to β-CD. Consequently, powerful electrochemical signal has been observed (Figure 1). Aptasensor sensitively respond to AFB1 in the ranges between 0.1 pg/mL and 10 ng/mL with lower LOD equal to 0.049 pg/mL by AC impedance detection. It has been found that aptasensor enjoyed acceptable reliability and selectivity and experienced a successful application for determining AFB1 in the real samples of the peanut oil with recovery in the range between 94.5% and 106.7% and intraassay RSD less than 11.51% [14].

Stepanova et al. assembled DNA sensors by consecutive deposition of thiacalix [4] arenes bearing oligolactic fragments, poly(ethylene imine) and DNA onto the glassy carbon electrode. The assembling of the layers was monitored with scanning electron microscopy, cyclic voltammetry and electrochemical impedance spectroscopy. The configuration of the thiacalix [4] arene core determined self-assembling of the polymeric species to the nano/micro particles with a size of 70–350 nm. Depending on the granulation, the coatings show the accumulation of a variety of DNA quantities, charges and internal pore volumes. These parameters were used to optimize the DNA sensors based on these coatings. Thus, doxorubicin was determined to have limits of detection of 0.01-nM (cone configuration), 0.05-nM (partial cone configuration), and 0.10-nM (1,3-alternate configuration of the macrocycle core). Substitution of native DNA with aptamer specific to aflatoxin M1 resulted in the detection of the toxin in the range of 20 to 200 ng/L (limit of detection 5 ng/L). The aptasensor was tested in spiked milk samples and showed a recovery of 80% and 85% for 20 and 50 ng/L of the aflatoxin M1, respectively [31]. Table 1, Table 2 and Table 3 shows the comparison in the efficiency of GCE based on aptasensors for detecting AFs.

### 4.2. SPE-Based Aptasensors

It is notable that novel encouraging opportunities for applying the electrochemical procedures for environmental analysis outside the centralized laboratories have been provided by emergence of the screen-printed technology. In fact, the screen-printing technology allows the mass production of the reproducible, affordable, and mechanically accurate solid strip electrodes. Moreover, it enjoys beneficial characteristics such as miniaturizing the corresponding device and simplified handling and manipulation in a disposable way. It also enables the biomolecules for immobilizing on the surface of electrode for fabricating the selective biosensors. Therefore, the sensing section has been considered the analyte-specific segment of the biosensors. Microorganisms, enzymes, antibodies, receptors and nucleic acids have a widespread application for constructing the SPE biosensors. These would be immobilized over the surface of the working electrode with a distinct nature like gold or carbon via absorption, micro-encapsulation, entrapment, cross-linking or the covalent attachment [43].

The investigation reported by Goud et al. provided an electrochemical aptasensor with the use of methylene blue (MB) redox probe labeled aptamer as a signaling element and functional graphene oxide (FGO) as a signal enlarging platform. Then, the functionalized graphene oxide cast on the SPCE and MB-tagged aptamer experienced a covalent immobilization on the SPCE via hexa-methylenediamine (HMDA) as a spacer through carbodiimide amide bonding chemistry. In addition, the AFB1 analyte molecule determination has been done by aptamer conjugated redox probe that undergoes or involves conformational changes in the complex structure of the aptamer consequent for binding AFB1. Their assay detected AFB1 in a linear range between 0.05 and 6.0 ng mL^−1^ with the very low LOD equal to 0.05 ng mL^−1^. Finally, this aptasensor has been experimented for screening the alcoholic beverage samples to determine AFB1 and acceptable recovery values have been reported [32].

In another study, Istamboulié et al. presented an aptasensor to detect AFM1 in the milk based on DNA-aptamer recognition as well as EIS detection. Therefore, a hexaethyleneglycol-modified 21-mer oligonucleotide has been immobilized on a SPCE via immobilizing carbodiimide upon the activation of diazonium of the sensing surface. In addition, EIS as well as CV in the presence of ferri/ferrocyanide redox probe have been utilized for characterizing all phases of the aptasensor design. Finally, aptamer-AFM1 interactions increased the level of the electron transfer resistance and allowed determining AFM1 in the buffer ranging between 2.0 and 150.0 ng L^−1^ (LOD equal to 1.15 ng L^−1^) [33].

**Figure 2:**
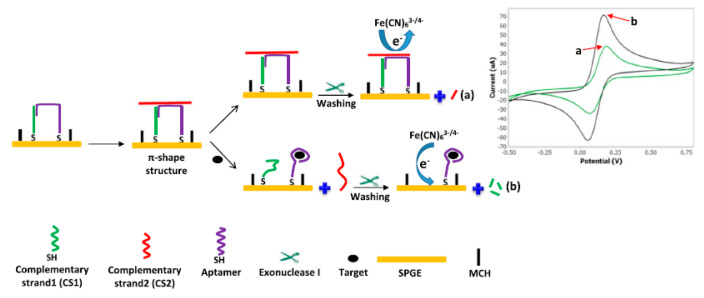
Construction procedure of the introduced π-shape electrochemical aptasensor for AFB1. (**a**) As seen, in the absence of AFB1, many sections of the π-shape structure remained intact and the redox mediator had no access to the electrode surface, resulting in a poor current signal. (**b**) Moreover, in the presence of AFB1, Apt attached to AFB1 and left the aptamer (Apt)-complementary strands (CSs). Exo I digested CS1, causing greater access of [Fe(CN)_6_] ^3−/4−^ to the electrode surface, producing a powerful current signal. Reprinted with permission from [4]. Copyright 2017, Elsevier.

One of the other investigations performed by Abnous et al. designed one of the precise electrochemical sensing strategies to determine AFB1 on the basis of aptamer (Apt)-complementary strands (CSs) complex, forming a π-shape structure on the electrode surface and exonuclease I (Exo I). It has been found that the presence of a π-shape structure as the double-layer physical barrier detects AFB1 with higher sensitivity. However, in the absence of AFB1, the π-shape structure has been intact; therefore, just a poor peak current has been registered. When AFB1 has been added, π-shape structure has been disassembled and a powerful current has been registered when Exo I have been added. The principle of the proposed electrochemical biosensor was based on the π-shape structure of Apt-CSs complex as a double-layer physical barrier for the access of [Fe(CN)_6_] ^3−/4−^ to the surface of gold electrode and Exo I-assisted signal amplification (Figure 2).

Ferro-/ferricyanide salts, such as hexacyanoferrate ([Fe(CN)_6_] ^3−/4-^) are commonly used as a redox probe in electrochemistry. In recent years, the incorporation of [Fe(CN)_6_] ^3−/4-^ into electrode fabrication has been widely explored. In a simplified scheme, the ferricyanide in the bulk solution will approach the working electrode where the reduction takes place, then ferrocyanide ions will also diffuse into the bulk solution. Thus, the electrical current is an appropriate measurement for the overall reaction rate, given that this is directly proportional to the amount of substance produced at each electrode, according to Randles–Sevcik equation.

Based on the optimum condition, electrochemical signals increased by AFB1 concentration enhanced with the dynamic ranges between 7 and 500 pg mL^−1^ with LOD equal to 2 pg/mL. Finally, Abnous et al. demonstrated aptasensor for analyzing the AFB1 spiked human serum and the grape juice samples with recovery ranging between 95.4% and 108.1% [4].

One of the other studies performed by Goud et al. presented a label-free electrochemical impedimetric aptasensor to determine AFB1. The researchers made a comparison between analytical functions of 2 aptamer sequences (seq. A and seq. B). Therefore, detection has been based on the recognition through the aptamer covalently bound as the compact monolayer on the SPCEs via the diazonium coupling reaction. Moreover, EIS has been used to quantify AFB1. According to the analyses, the dynamic quantification ranged between 0.125 and 16 ng mL^−1^ in the 2 kinds of the aptamer sequences whereas LOD, respectively was equal to 0.12 and 0.25 ng/mL for seq. A and seq. B. In addition, for the real sample utilizations, these new aptasensors have been approved in the wine and beer samples and acceptable level of recoveries ranging between 92% and 102% have been recorded for AFB1 determination [34].

Pandey et al. presented an electrochemical aptasensor to determine the trace level of AFM1. Therefore, aptamer has been immobilized on the screen-printed gold electrode via the sequentially layering dithiodipropionic acid, streptavidin and biotinylated-tetra ethylene glycol-aptamer results showing the inverse correlation of the peak current in the square wave voltammogram with AFM1 logarithmic concentration. Moreover, the sensor dynamic range is equal to 1.0 and 10^5^ ppt AFM1 [35].

In addition, Jalalian et al. designed an electrochemical aptasensor to determine AFM1 based on the hairpin-shaped structure of AFM1 aptamer (Apt), CS and AuNPs. According to their analyses, conformational changes in the hairpin structure of Apt in the absence and presence of AFM1 as well as the Au NPs with negative charges detect AFM1 with higher selectivity and sensitivity. Moreover, in the absence of AFM1, hairpin structure of the Apt remains intact. Thus, a poor peak current has been achieved. Nonetheless, adding AFM1 results in disassembling the Apt hairpin structure. Therefore, the CS modified Au NPs closely reach the screen-printed gold electrode surface and a powerful current signal has been registered by adding the MB as the redox agent. In addition, aptasensor could determine AFM1 with the LOD equal to 0.9 ng L^−1^. Ultimately, this new aptasensor experiences a successful application to determine AFM1 in the real samples, such as serum and milk samples [36].

**Figure 3:**
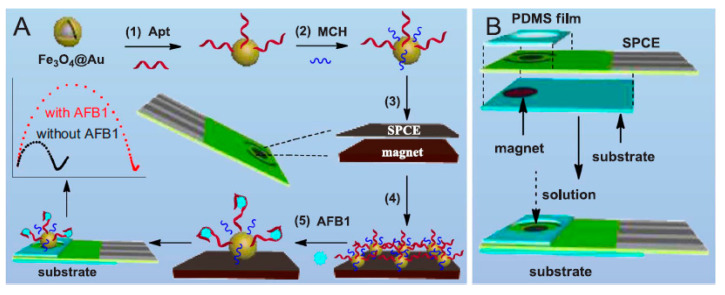
A schema of the fabrication procedure of (**A**) the magnetically assembled aptasensor and its working principle for determination of AFB1, and (**B**) the aptasensing instrument. Reprinted with permission from [37]. Copyright 2018, Elsevier.

Moreover, Wang et al. proposed a magnetically assembled aptasensing instrument to determine label-free AFB1 with a disposable SPCE covered with the poly-dimethylsiloxane (PDMS) film as a microelectrolytic cell. Therefore, researchers initially procured the magnetically controlled bio-probes via immobilizing the thiolate aptamers over the Fe_3_O_4_@Au magnetic beads which are quickly assembled on the SPCE working electrode within ten seconds with a magnet at the opposite side. Then, PDMS film containing a centered hole was covered on the surface of SPCE surface for achieving more flexible and functional electrochemical measurements. It is notable that Wang et al. introduced a label free aptasensor for sensitively and selectively determining AFB1 with EIS after biorecognition between the aptamers and targets (Figure 3). Analyses indicates wider linear ranges between 20.0 pg/mL and 50.0 ng/mL and the LOD equal to 15.0 pg/mL (S/N = 3) for this sensor. Finally, the presented sensing method represents an encouraging option for controlling the quality and safety of the foodstuffs [37].

Furthermore, Aissa et al. reported designing a label-free aptasensor on the basis of silicon NPs (SiNPs) for ultra-sensitively detecting AFM1 in the milk. Since the silicon nanomaterials are popular for their great capacitive power, researchers utilize them for developing a new capacitive transduction system on the basis of the electrochemical capacitance spectroscopy. Such an approach depends on the modifications in the redox capacitance signal because of the surface-tethered ferrocene film via EIS measurements without employing an exterior redox probe. According to the outputs, the redox capacitance variations have a good correlation with the enhancing concentrations of AFM1 in the linear range between 10.0 and 500.0 fmol L^−1^. Moreover, this aptasensor reaches very low LODs and limit of quantification equal to 4.53 fM and 14.95 fM. Finally, their platform demonstrated the increased selectivity towards the target analyte and utilized for quantifying very low concentrations of AFM1 in the commercial pasteurized milk [38].

One of the studies reports an electrochemical enzyme linked oligonucleotide array for obtaining a simplified and fast multi-detection of AFB1. Therefore, assaying has been done regarding a competitive format and disposable screen-printed cells (SPCs). Initially, poly (aniline-anthranilic acid) copolymer (PANI-PAA) was electrodeposited on the graphite screen-printed working electrodes with CV. Afterwards, covalent binding on PANI-PAA copolymer was used to immobilize the Aflatoxin B1 conjugated with bovine serum albumin (AFB1-BSA). Following the affinity reaction between biotinylated DNA-aptamer (apt-BIO) and AFB1, the solution was dropped on the modified SPCs and the competition was made. Next, the biotinylated complexes established on the surface of the sensor was coupled with a streptavidin–alkaline phosphatase conjugate. Consequently, 1-naphthyl phosphate was utilized as the enzymatic substrate; DPV was used to detect the electroactive product. Notably, the response of the enzyme-linked oligonucleotide assay is signal–off, based on the competitive format. Therefore, a dose–response curve is observed from 0.1 to 10 ng/mL and LOD equal to 0.086 ng mL^−1^. Ultimately, the introductory tests in maize flour samples spiked with AFB1 have been done [39].

Another study conducted by Zejli et al. presented one of the electrochemical aptasensors with MB as the signaling fragment and poly thiophene-3-carboxylic acid (PT3C) as a signal enlarging platform. Initially, they screened PT3C over the SPCE interface and thus immobilization of MB-tagged aptamer was conducted on the SPCE with the HMDA as a spacer through the carbodiimide amide bonding chemistry. In fact, the researchers selected AFB1 as the model analyte for testing their aptasensing platform. Results show acceptable dynamic ranges between 2.5 and 30.0 ng L^−1^ for AFB1 with the LOD equal to 1.6 ng L^−1^. In addition, a reasonable reproducibility has been recorded with RSD% equal to 3.21. It has been found that such an aptasensor is utilized in the coffee samples so that satisfactory recoveries are observed in ranges between 88.2% and 93.3%, reflecting the new aptasensor efficacy for the coffee samples [7].

### 4.3. Gold Electrode-Based Aptasensors

As stated in the studies, the gold electrode is considered to have biocompatibility, lower capacitance and lower electrical resistance. Moreover, the chemical state of the gold electrode is regarded nearly stable so that its structure can be monitored via choosing the proper experimental condition. In addition, the gold electrode is represented to be an adequate candidate for examinations on the interactions between biomolecule and electrode surface [44].

In their study, Dinckaya et al. described fabrication and utilization process of a novel DNA biosensor to determine AFM1. For immobilizing a thiol-modified single stranded DNA (ss-HSDNA) probe, which particularly bound the aflatoxin M1, a self-assembled mono-layer of the AuNPs and cysteamine over the SAM was procured on the gold electrodes. In addition. CV and EIS procedures have been used to control the assembly procedures of AuNPs via a layer-by-layer method, cysteamine, and ss-HSDNA. Notably, the K_3_ [Fe(CN)_6_]/K_4_ [Fe(CN)_6_] solution was utilized as a redox probe to do electrochemical measurement. Therefore, biosensor linearly responds to AFM1 in a concentration ranging between 1 and 14 ng mL^−1^ with the standard deviation equal to ± 0.36 ng mL^−1^. Ultimately, this biosensor was utilized for some real milk samples [13].

Moreover, Castillo et al. introduced an aptamer-based biosensor to determine AFB1 as a pollutant in the foodstuff. Their sensor was assembled in a multi-layer framework, which utilized EIS and CV to acquire the signal response through the redox indicators of K[Fe(CN)_6_] ^3-/4–^. In addition, the poly (amidoamine) dendrimers of fourth generation (PAMAM G4) immobilized on the gold electrode coated by cystamine was utilized to attach the single stranded amino-modified DNA aptamers specific to the AFB1. Then, a comparison was made between cystamine dendrimers (Cys-PAMAM) layers and other immobilization platforms like 11-mercaptoundecanoic acid (MUA), 11-mercaptoundecanoic acid dendrimers (MUA-PAMAM) and cystamine (Cys), which was the initial method with the highest level of suitability to generate the reproducible and sensitive signal in a concentration range of 0.1 and 10-nM AFB1. Finally, validity of this sensor has been confirmed in the polluted peanuts extract and in the spiked samples of the peanuts-corn snacks. Moreover, the sensing response was assessed and for the matrix impact [40].

In addition, Zheng et al. investigated the development of an electrochemical aptasensor for the AFB1 trace determination by employing an aptamer as the recognition unit and selecting telomerase and EXOIII-based two round signal amplification approach as the signal enhancement units. Therefore, researchers utilized telomerase amplification for elongating the ssDNA probes over AuNPs surface; the signal response of the signal–off model electrochemical aptasensor became larger. Afterwards, EXOIII amplification was applied for hydrolyzing the 3′-end of the dsDNA following the target AFB1 recognition which resulted in releasing the bounded AFB1 in the sensing system, wherein it involved in the next recognition-sensing cycle. Thus, with this two-round signal amplified electrochemical aptasensor, the targeted AFB1 was substantially measured at the trace concentration with very good LOD equal to 0.6 × 10^−4^ ppt and met specificity because of the very good affinity of the aptamer against AFB1. However, on the basis of the introduced two-round signal amplification approach, the sensing range as well as LOD is highly enhanced [20].

Furthermore, Karapetis et al. comparatively analyzed the sensitivity of the aptamer-based biosensors to determine the mycotoxin AFM1 with regard to the immobilization procedure of the DNA aptamers as well as determination technique. Therefore, the label-free EIS and DPV for the ferrocene labeled neutravidin layers were utilized. In addition, immobilization of the amino-modified DNA aptamers was performed at the surface of poly-amidoamine dendrimers (PAMAM) of the 4th generation or the biotin-modified aptamers have been immobilized at the neutravidin layer chemisorbed at the surface of the gold. For the first case, LOD is equal to 8.47 ng L^−1^. However, in the second approach, LOD is equal to 8.62 ng L^−1^ which is lower than the permitted limits of AFM1 in the milk as well as the milk products. Finally, validity of the aptasensors was confirmed in the spiked milk samples with acceptable recovery superior to 78% [41].

**Figure 4:**
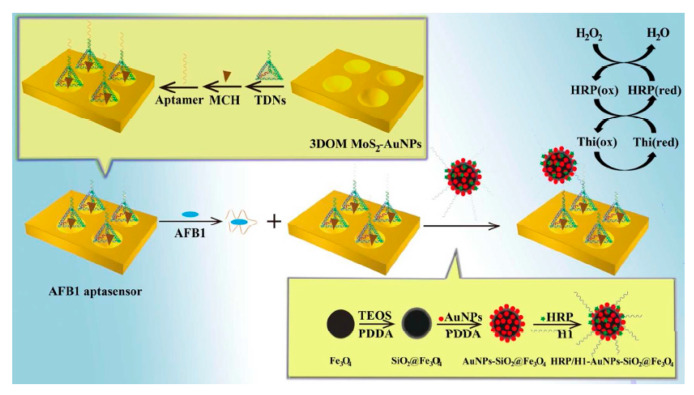
Schema of the protocol of electrochemical AFB1 aptasensor. Reprinted with permission from [42]. Copyright 2018, ACS.

Additionally, Peng et al. developed an AFB1 electrochemical aptasensor on the basis of tetrahedral DNA nano-structures (TDNs) immobilized 3D ordered macroporous MoS_2_-AuNPs hybrid (3DOM MoS_2_-AuNPs) recognition interface and horseradish peroxidase (HRP) functionalized magnetic signal amplifier (Figure 4). Notably, for significant enhancement in the recognition efficacy, aptasensor stability and sensitivity, AFB1 aptamer-incorporated TDNs were ingeniously combined with 3DOM MoS_2_-AuNPs film in order to make a sensing interface. In this way, aptamers released from the surface of the gold electrode after their reaction with AFB1 and hybridization-free TDNs were established. Therefore, biocomposite of DNA helper strands (H1)/HRP-functionalized AuNPs-SiO_2_@Fe_3_O_4_ nanospheres combined with hybridization-free TDNs because of TDNs and H1 hybridization. In fact, if there is higher AFB1 in the solution, greater H1/HRP-AuNPs-SiO_2_@Fe_3_O_4_ would be combined into 3DOM MoS_2_-AuNPs surface. According to the analyses, the current response from the HRP catalysis reduction of H_2_O_2_ by thionine (Thi) as the electrochemical probe has been in proportionate to AFB1 concentration. Based on the optimized condition, aptasensor exhibited specificity for AFB1 and achieved an acceptable linear range from 0.1 fg mL^−1^ to 0.1 μg mL^−1^ and LOD equal to 0.01 fg mL^−1^. Finally, this new aptasensor was utilized to determine AFB1 contents in the rice and wheat powder samples [42,45,46,47,48].

## 5. Conclusions

As populations increase worldwide, it is hard to control food contamination in human lives. Hence, it is crucial to provide warning mechanisms to properly recognize food contamination and suppress toxins such as aflatoxins (AFB1 and AFM1). It is highly advised to design sensitive and reliable techniques because of the decreased amounts of such poisonous metabolites. However, numerous publications addressed advancements to develop aptamer for improving the detection processes. Therefore, researchers greatly considered the aptamer-based biosensors due to their accuracy and rapidness in detecting AFM1 and AFB1 as the food contaminants. Moreover, specific characteristics of aptamers like simplified synthesis, higher specificity, stability and sensitivity in diverse conditions make them encouraging options to design different sensing platforms. Despite diverse actions taken for confirming effectiveness of different aptamers in sensing processes, many efforts should be made for AFB1 and AFM1 aptasensors for feasible diagnosis.

Therefore, one of the potentially increasing fields addresses advances in the aptamer immobilization approaches particularly in the combination with the nanomaterials. Such limitations demonstrated the existence of encouraging superior opportunities in the area of aptasensors. Hence, further studies should emphasize designing aptamers against the unstudied target analytes and their integration in reliable and popular electrochemical platform for analyses.

## Figures and Tables

**Table 1:** Comparison of the efficiency of glassy carbon electrode (GCE)-based aptasensors to detect aflatoxins (AFs).

Electrode Materials	Analyte	Analytical Methods	LOD	Linear Range	Real Samples	Aptamer Length	Sequences	Ref.
Neutral red-thiacalix [4] arene A-DNA aptamer	AFB1	CV	0.1 nM	0.1–100 nM	peanuts, cashew nuts, white wine and soy sauce samples	50	5′-GTT GGG CAC GTG TTG TCT CTC TGT GTC TCG TGC CCT TCG CTA GGC CCA CA-3′	[1]
RGO/MoS_2_/PANI@AuNPs	AFB1	DPV	0.002 fg/mL	0.01 fg/mL–1.0 fg/mL	Wine samples	50	5′-GTT GGG CAC GTG TTG TCT CTC TGT GTC TCG TGC CCT TCG CTA GGC CCA CA-3′	[29]
poly NR–P [5] A COOH layer modified with the aptamer	AFM1	EIS	0.5 ng/L	5–120 ng/L	sheep and cow milk samples	21	5′-ACT GCT AGA GAT TTT CC CAT-3′	[30]
Thiacalix [4] arenes bearing oligolactic fragments, poly(ethylene imine) and DNA	AFM1	EIS	5.0 ng/L	20.0–200.0 ng/L	Milk samples	21	50-NH2-ACT GCT AGA GAT TTT CCA CAT-30	[31]

**Table 2:** Comparison of the efficiency of SPE-based aptasensors to detect AFs.

Electrode Materials	Analyte	Analytical Methods	LOD	Linear Range	Real Samples	Aptamer Length	Sequences	Ref.
Apt-complementary strands complex	AFB1	DPV	2.0 pg/mL	7.0–500.0 pg/mL	human serum and grape juice samples	–	–	[4]
Graphene/methylene blue tagged aptamer	AFB1	DPV	0.05 ng mL^−1^	0.05–6.0 ng mL^−1^	Alcoholic beverage samples (beer and wine)	35	50-TGGGGTTTGGTGGGTGGTGTACGGGCAGG-30	[32]
AFM1-Aptamer	AFM1	Impedimetric	1.15 ng/L	2.0–150.0 ng/L	Milk samples (raw milk, micro-filtered full-fat milk, pasteurized full-fat milk and pasteurized skimmed milk)	21	5′-ACT-GCT- AGA-GAT-TTT-CCA-CAT-3′	[33]
diazonium/Aptamer (two aptamers sequences A and B)	AFB	EIS	0.12 ng/mL	0.125–16.0 ng/mL	Wine and beer samples	35	5′TGGGGTTTTGGTGGCGGGTGGTGTACGGGCAGGG-3′	[34]
biotinylated-TEG-aptamer	AFM1	SWV	–	1.0–105 ppt	–	72	5′ATCCGTCAACCTGCTCTGACGCTGGGGTCGACCCGGAGAAATGCATTCCCTGTGGTGTTGGCTCCCGAT-TEG Biotin3′	[35]
CS-modified AuNPs/Apt	AFM1	DPV	0.9 ng/L	2.0–600.0 ng/L	Serum and milk samples	–	–	[36]
Fe3O4@Au-Apt	AFB1	EIS	15.0 pg/mL	20.0 pg/mL−50.0 ng/mL	Peanut samples	50	5′-GTT GGG CAC GTG TTG TCT CTC TGT GTC TCG TGC CCT TCG CTA GGC CCA CA-SH-3′	[37]
anti-AFM1/Fc/SiNPs-PpPD	AFM1	Electrochemical capacitance spectroscopy (ECS)	4.53 fM	10.0–500.0 fmol/L	Commercial pasteurized milk sample	21	5′-ACT GCT AGA GAT TTT CCA CAT-3′	[38]
apt-BIO/AFB1-BSA/PANI-PAA	AFB1	DPV	0.086 ng/mL	0.1–10.0 ng/mL	Maize flour samples	50	5′-(biotin)-TEG(triethylene glycol)- GTT GGG CAC GTG TTG TCT CTC TGT GTC TCG TGC CCT TCG CTA GGC CCA CA-3′	[39]

**Table 3:** Comparison of the efficiency of gold electrode-based aptasensors to detect AFs.

Electrode Materials	Analyte	Analytical Methods	LOD	Linear Range	Real Samples	Aptamer Length	Aptamer Length	Ref.
21-mer ss-HSDNA	AFM1	EIS	–	1.0–14.0 ng/mL	Milk sample	21	ss-HSDNA (5_-thiol-(CH2)6 ACT GCTAGA GATTTT CCA CAT-3_)	[13]
telomerase primer—AuNPs-c-DNA/MB	AFB1	SWV	0.6 × 10^−4^ ppt	0.0001–100.0 ppt	Corn samples	50	5′- GTTGGGC AC GTGTT GTCTCTC TGTGTCT CGTGCCC TTCGCTA GGCCCA CA-(CH2)6-SH-3′	[20]
Aptamers on dendrimers	AFB1	EIS, CV	0.4 nM	0.1–10.0 nM	Peanut samples	50	NH2-5′-GTTGGG CACGTG TTGTCTC TC TGTGTC TCGTGCCCTTCG CTAGGCC CA CA-3′	[40]
Biotinylated aptamers immobilized at neutravidin layer modified by ferrocene	AFM1	DPV	8.47 ng/L	15–120 ng/L	Milk samples	21	50-ACT GCT AGA GAT TTT CCA CAT-30 (APT1)	[41]
			8.52 ng/L			35	50-TTT TTT TTT TTT TTT ACT GCT AGA GATTTT CCACAT-30 (APT2	
			8.64 ng/L			50	50-GTT GGG CAC GTG TTG TCT CTC TGT GTC TCG TGC CCT TCG CTA GGCCC CA-30 (APT3)	
3DOM MoS2-AuNPs,HRP/AuNPs-SiO2@Fe3O4	AFB1	DPV	0.01 fg/mL	0.1 fg/mL–0.1 μg/mL	Rice and wheat powder samples	–	–	[42]
